# Stimulus-Response Compatibility Effect in the Near-Far Dimension: A Developmental Study

**DOI:** 10.3389/fpsyg.2016.01169

**Published:** 2016-08-05

**Authors:** Aurélien Richez, Gerard Olivier, Yann Coello

**Affiliations:** ^1^CNRS, UMR 9193 – SCALab – Sciences Cognitives et Sciences Affectives, Université de LilleLille, France; ^2^Laboratoire Interdisciplinaire Récits Cultures Et Sociétés, University of Nice Sophia AntipolisNice, France

**Keywords:** development, stimulus-response compatibility, reaching, reaction time, movement time

## Abstract

The present study investigates the developmental aspect of stimulus-response compatibility (SRC) effect in 8–11-years-old children. The task consisted in manually responding to the color of a pawn presented on a chessboard at different distances. Manual responses were provided by reaching a proximal or distal location depending on the color of the stimulus. We found that reaction time was affected by the conflict generated by the response suggested by the location of the stimulus and the response required according to its color. This was not the case for movement time despite we found a higher rate of long duration movements in the incongruent than in the congruent spatial condition. The SRC effect was, however, observed in children older than 10 years old. These findings provide additional evidence for a reorganization of the perceptual system during the period of 8–10 years, integrating progressively multimodal information and preparing more efficiently the body to act in the environment.

## Introduction

Since the last century, the role of action in perception and cognition as been widely discussed in philosophy (e.g., Merleau-Ponty, 1945; Hurley, 2002; Noë, 2004), psychology (e.g., James, 1890; Piaget and Inhelder, 1969; Gibson, 1979; Coello and Fischer, 2015) as well as in neurosciences (e.g., Jeannerod, 2006; Rizzolatti and Sinigaglia, 2008). Common to the different theories is the shared idea that perception, action and cognition coevolve through the active exploration of the body and the environment, and contribute through their interaction to knowledge acquisition and retention (Hommel et al., 2001; Chemero, 2003; Barsalou, 2008). In agreement with this view, developmental studies have emphasized the importance of very early interactions with the environment for the ontogenetic development of perceptual (Held and Hein, 1963; Fine et al., 2003) and cognitive abilities (e.g., Wallon, 1945; Piaget and Inhelder, 1969; Thelen and Smith, 1994). On the basis of observations and experimental data, these pioneer researchers have furthermore highlighted the non-linear trajectory of sensorimotor and cognitive developments, characterized by significant quantitative and qualitative changes taking place during the ontogenesis. Within this framework, gradual improvements in perceptual, motor and cognitive abilities through the ontogenetic development were interpreted as relying on structural and functional maturation of the nervous system (Gesell, 1929; Carmichael, 1946; McGraw, 1946), as well as on the multi-dimensional and dynamical aspect of brain mechanisms relating to behaviors (Greenough et al., 1987; Fischer and Bidell, 1998; Segalowitz, 2002), leading progressively to more efficient processing and integration of multimodal information (Choudhury et al., 2007; Gori et al., 2008; Nardini et al., 2010).

Putting development under careful scrutiny has emphasized a particular critical period around the age of 8–10 years, characterized by strong reorganization of motor, perceptual and cognitive activities. Indeed, even if performances are still not equivalent to adults or even adolescents (Choudhury et al., 2007), 8–10 years-old children behave differently than younger children in that they base their perception on multimodal sensory integration (Gori et al., 2008; Nardini et al., 2010), use more efficient predictive mechanisms in their motor performance (Thibaut and Toussaint, 2010) and reveal better flexibility and inhibition capacities in cognitive control (Houdé and Guichart, 2001). Considering the perception of objects’ orientation and size for instance, 5–6 years-old children exhibit a clear dominance of one modality upon the other, basing their perception on single (visual or haptic) sensory information. In contrast, 8–10 years children integrate visuo-haptic information in their perceptual estimates (Gori et al., 2008), in accordance with optimal integration statistical models characterizing adults’ performances (Ernst and Banks, 2002; see also Nardini et al., 2008, 2010 for similar findings with multidimensional integration of unimodal sensory information). During this period of age, motor control also improves significantly (Assaiante and Amblard, 1995), with more appropriate anticipatory control of posture (Girolami et al., 2010) and object-directed motor actions (Forssberg et al., 1992), as observed in eye-hand coordination (Ego et al., 2016), simple motor tasks such as moving a lever (Bard et al., 1990), or more complex motor tasks such as writing or drawing (Meulenbroek and Van Galen, 1988; Lange-Küttner, 2009). From 8 to 10 years, children show influence of the terminal state of the body during the programming stage of object-directed motor actions (end-state-comfort-effect, Adalbjornsson et al., 2008; Thibaut and Toussaint, 2010) as well as a decrease of movement speed (Bard et al., 1990), suggesting a reorganization of motor planning and guiding strategies based on the refinement of internal models supporting motor skills and predictive behaviors (Thibaut and Toussaint, 2010; Ego et al., 2016). This is also at this age that the capacity to relate sensorimotor and visual information appears a major determinant of perceptual performances, as evidenced, for instance, by the increase accuracy in visually detecting what is reachable with the arm (Richez and Coello, 2015).

As visuo-motor reorganization appears to be spread across a large span of abilities implying the integration of sensorimotor information, we expected developmental changes during the period between 8–10 years to influence the effect of stimulus-response compatibility (SRC). SRC effect is characterized by a faster and more accurate performance when the location of a visual stimulus is compatible with the location of the motor response provided according to a specific attribute of the stimulus. The classical example of such effect is the Simon effect (Simon and Rudell, 1967; Simon and Small, 1969), which corresponds to the situation where participants must respond to a left or right visual stimulus with one hand (e.g., right) when the stimulus has one color (e.g., red), and with the other hand (e.g., left) when the stimulus has a different color (e.g., green), regardless the location of the visual stimulus. Although the participants are not supposed to take into account the position of the visual stimulus, they usually react faster when the color of the stimulus that appears on one side corresponds to the motor response provided on the same side (congruent trials), compared to the motor response provided on the opposite side (incongruent trials). Following this seminal study, the Simon effect has been used as a valuable tool to study multimodal perception as well as the relation between perception and action (Tucker and Ellis, 1998; Buetti and Kerzel, 2009; Hommel, 2011). Furthermore, Simon’s followers have adapted the original paradigm by changing intrinsic and extrinsic features of the stimuli while generally retaining the left-right motor responses (e.g., Hommel, 2011 for a review). To account for SRC effect as a whole, several authors have suggested the existence of two distinct routes from the stimulus to the response, namely a direct and an indirect route (e.g., Kornblum, 1994; Eimer et al., 1995). The first one, the direct route, characterizes an automatic activation of the motor response in relation to the location of the stimulus, and a facilitation effect when both the stimulus and the response features spatially overlap. The second one, the indirect route, links stimulus and response through an arbitrary relationship depending on the experimental instructions. In this respect, Hommel et al. (2001) suggested that the overlap between the perceptual and motor codes within a common and amodal coding system of the various features of an object originates the SRC effect. Accordingly, SRC effect does not depend of the relevant or irrelevant nature of the stimulus-response features in relation to the task, but simply the presence of an overlap between perceptual and motor dimensions (Kornblum, 1994; Hommel, 1997). In agreement with this, SRC effect is not restricted to the right-left dichotomy and several studies have shown that the effect can be extended to the near-far (e.g., Olivier, 2006; Coutte et al., 2015) as well as the up-down (e.g., Cho and Proctor, 2002; Meiran, 2005) spatial dimensions. Furthermore, SRC effect is thought to affect response selection as well as the subsequent programming and execution stages of motor responses (Buetti and Kerzel, 2008, 2009; Coutte et al., 2015).

In order to probe the developmental aspect of the relation between perception and action, we implemented a SRC task in a population of children extending from 8-years to over 11-years, a period characterized by major developmental changes related in particular to the integration of multisensory and motor information. In relation to the literature summarized above, we expected to observe the SRC effect with a classical pattern of facilitation for congruent relationship between perception and action, but predominantly in older children when the ability to optimally combine multisensory and motor information arises. In line with Buetti and Kerzel (2008), we expected also an improvement of children performances in terms of lower reaction time (RT) and movement time (MT) in the congruent condition. In our implementation of the SRC paradigm, children were exposed to a chessboard with one black or white pawn presented either at a proximal or distal location, as previously used by Coutte et al. (2015) to evaluate the SRC effect in the near-far dimension. The task was to respond to the color of a chess pawn by responding with the right hand toward either a proximal or distal location, regardless of the proximal-distal position of the pawn. We manipulated thus the compatibility of the perceived distance of the stimulus (the irrelevant feature) and the response distance during a simple color discrimination task (the relevant feature).

## Materials and Methods

### Participants

A total of 120 children (age range between 8 years 1 month and 12 years 3 months; *M* = 10 years, *SD* = 1 year) were recruited from French public elementary schools (grades 4–7). The population was then divided into 4 groups, according to parti cipant’s age: 8-years-old (*N* = 30; *M* = 8 years 8 months, *SD* = 3 months), 9-years-old (*N* = 30; *M* = 9 years 6 months, *SD* = 3 months), 10-years-old (*n* = 30; *M* = 10 years 6 months, *SD* = 3 months), 11-years-old (*N* = 30; *M* = 11 years 6 months, *SD* = 4 months).

During the recruitment, consents were obtained from the French National Education Services as well as from the children and their parents. Although practical information was provided to the children, none of them was informed prior to the experimental session about the aim of the study. Full explanations were nonetheless given at the end of the experiment and results were shown to the children. None of the participants had previous record of neurological or psychiatric disorders, or any kind of motor impairment. All participants were right-handed and had a normal (or corrected to normal) vision.

### Apparatus and Stimuli

**FIGURE 1:**
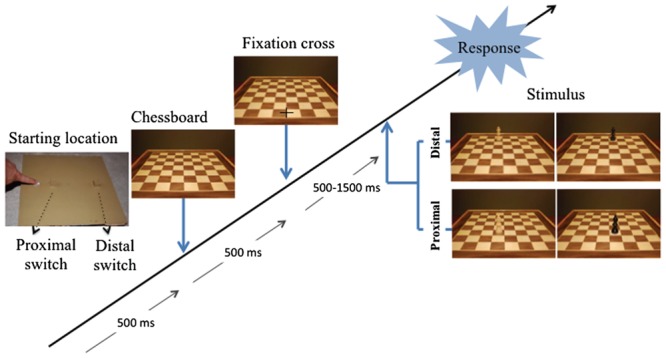
**Experimental set up and time sequence of the presentation of the stimuli used in the experiment**.

The experimental apparatus consisted in a computer screen and a response device. The computer screen (22′′, 100 Hz) was placed vertically on a table in front of the participant at a distance of about 57 cm. The response device was placed on the table between the participant and the computer screen. It consisted of 3 hand-operated switches fixed on a 30-cm-square wooden panel. The 3 switches were aligned with the participant’s mid-body sagittal axis. The closest switch (“starting location switch”) was a flat 2-cm-square and was situated 1 cm from the proximal edge of the panel. It could be operated by a simple contact or release using the forefinger. The 2 other switches were cubic sensors (2 × 2 × 2 cm), the first one was placed at 10 cm and the second one at 25 cm from the starting location. Both of them needed a precision grip using the thumb and the forefinger to be operated (see **Figure 1**).

Nine colored pictures (1280 × 857 pixel, resolution 300 dpi) representing a square-chessboard (30 cm × 30 cm) were used as stimuli. The pictures reproduced the visual perspective that a chess player would have when facing an actual chessboard. One picture represented an empty chessboard, the other pictures represented a chessboard with one pawn. The pawn was placed on one of the squares of the 2 central columns. Four possible locations were selected: 2 proximal locations (3rd row) and 2 distal locations (7th row). Half of the pawns were black and the other half was white. Four other pictures were used for the training session. They represented black and white pawns placed at a central location relatively to the other stimuli (5th row). The presence of a black pawn on a white squares, or vice versa, was counterbalanced.

### Procedure

After having been informed about the purpose and conduct of the experiment, the participants were invited to sit in front of the apparatus and to perform a short training session following the same procedure as for the forthcoming experimental session. The actual experiment started when the participants were able to provide motor responses accurately and fluently without the necessity for visual guidance of the moving hand. Before starting the experiment, the participants were allowed to adjust their chair and position to ensure that they could easily perform the proximal and distal manual responses. The proximal response consisted in grasping the nearest switch whereas the distal response consisted in grasping the farthest one. The participants provided their responses with the right hand, the left one remaining in a resting position on the table during the whole experimental session. Half of the participants were instructed to perform a proximal response in the presence of a white stimulus and a distal response in the presence of a black stimulus. The other half received the opposite instruction. At the beginning of each trial, a pictogram was displayed to the participants to indicate that they have to place their hand on the starting position. 500 ms after having reached the starting position, the photograph of an empty chessboard was displayed. Then, following a constant period of 500 ms, a fixation cross appeared at a location on the chessboard that was presented as corresponding to the location of the starting location for the hand on the response device (1 cm from the proximal edge of the chessboard, along the mid-body sagittal plane). After a random period of 500–1500 ms, the fixation cross disappeared and the stimulus (the chessboard with a pawn) was displayed. The participants were instructed to respond as fast and accurately as possible, the stimuli remaining visible until the participants provided their response. An error signal was displayed when the participants anticipated their response (i.e., lift their finger before the apparition of the stimulus) or produced a wrong answer. The whole experimental session was accomplished within one block of trials, including five presentations of the black and white pawns at a proximal and distal location in each column (random order), for a total of 40 trials. All trials with error or anticipation, as well as those with a response time below 200 ms or above 1000 ms, were repeated at the end of the block of trials. The experimental design corresponded thus to the equation P_15_*I_2_*A_4_*<R_2_*S_2_>, with P for Participants, I for Instruction (white pawn for proximal response or white pawn for distal response), A for Age group (8, 9, 10, 11), R for manual Response (proximal or distal), S for Stimulus location on the chessboard (proximal or distal).

### Data Recording and Analysis

Both stimulus display and response recording were under the control of Matlab software (R2008b, mathworks). In each trial, the response provided by the participants as well as RT and MT were recorded by the computer. RTs were computed as the time elapsed between the presentation of the stimulus and response initiation (the lift of the switch at the starting position). Movement times were computed as the time elapsed between the release of the starting position switch and the grasping of one of the response switches. In order to precisely catch the temporal dynamics of the individual performances, we analyzed the asymmetry of the distribution of the temporal measures, thought to highlight the variability associated with developmental acquisitions (Immordino-Yang and Fischer, 2007; Rose and Fischer, 2008), by fitting the data with an ex-Gaussian fit procedure (Ratcliff and Murdock, 1976; Lacouture and Cousineau, 2008). The ex-Gaussian distribution is a convolution of a normal and an exponential distribution. The probability density function of the ex-Gaussian fit is given by the following equation:

(1) $$\begin{array}{c} f\left(x|\mu ,\sigma ,\tau \right)=\frac{1}{\tau }\exp(\frac{\mu }{\tau }+\frac{\sigma^{2}}{{2\tau }^{2}}-\frac{x}{\tau })\Phi \left(\frac{x-\mu -\sigma^{2}/\tau }{\sigma }\right) \end{array}$$

where Φ represents the value of the cumulative density of the Gaussian distribution.

Ex-Gaussian fit procedure was performed using the MATLAB toolbox “DISTRIB” function, in accordance with Lacouture and Cousineau (2008). The ex-Gaussian function applied to empirical RT and MT data provided estimates for three independent parameters: mu, sigma, and tau. Mu (μ) represents the mean of the normal component and mainly reflects the average performance. Sigma (σ) corresponds to the *SD* of the normal component and indicates the variability of the performances. Tau (τ) corresponds to both the mean and the standard deviation of the exponential component and reflects extremes in performance and thus the asymmetry of the function (a greater τ means a higher amount of long responses compared to short responses, Matzke and Wagenmakers, 2009).

As the instruction factors did not show any significant effect, a 2 × 2 × 2 analysis of Variance (ANOVA) was run on all dependant variables with *Target distance (proximal, distal)* and *Manual response (proximal, distal)* as within-subject factors and *Age group (8, 9, 10, 11)* as between-subjects factor.

## Results

### Reaction Time

**FIGURE 2:**
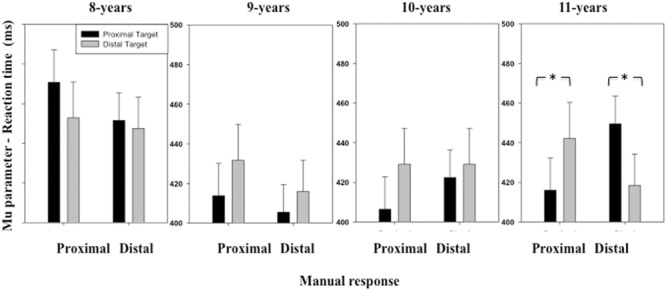
**Mean μ parameter of the ex-Gaussian distribution (reaction time, RT) as a function of Age, Target location (proximal, distal), and Response (proximal, distal).** Error bars represent standard errors. The stars indicate significant differences.

The ANOVA performed on the μ parameter of the RT distribution revealed a significant interaction between *Stimulus location* and *Manual response* [*F*(1,116) = 5.07, *p* = 0.02] (see **Figure 2**). Overall, when children were presented with a proximal stimulus, RT was shorter when they grasped the proximal (*M* = 426 ms, *SD* = 91 ms) than the distal switch (*M* = 432 ms, *SD* = 79 ms). When the children were presented with a distal stimulus, RT was shorter when they grasped the distal (*M* = 426 ms, *SD* = 85 ms) than the proximal switch (*M* = 438 ms, *SD* = 97 ms). The ANOVA also revealed an interaction between these two factors and *Age group* [*F*(3,116) = 3.48, *p* = 0.01]. Contrasting the data obtained in the different age groups showed that the *stimulus location* and *manual response* interaction was not significant for the younger children [*F*(1,29) = 0.42, *p* > 0.05 for age group 8; *F*(1,29) = 0.55, *p* > 0.05 for age group 9], emerged as a statistical trend toward significance for the 10-years-old children [*F*(1,29) = 1.98, *p* = 0.16], and was significant for the oldest children [*F*(1, 29) = 14.13, *p* < 0.001 for age group 11]. For the 11-years old children, we observed the classic pattern of shorter RTs for congruent trials than incongruent ones as observed in adults, namely, RTs significantly increased in the incongruent response condition when the target was both at the proximal [*t*(29) = 2.23, *p* = 0.01] or distal [*t*(29) = 1.70, *p* = 0.04] location.

**FIGURE 3:**
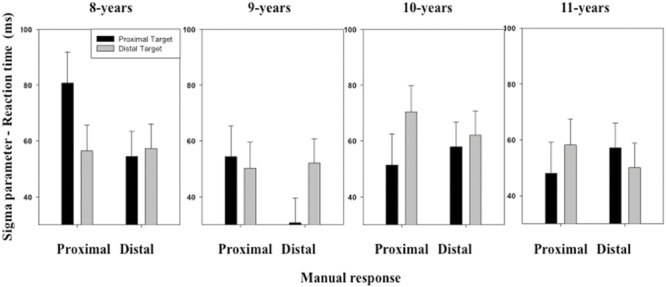
**Mean σ parameter of the ex-Gaussian distribution (RT) as a function of Age, Target location (proximal, distal), and Response (proximal, distal).** Error bars represent standard errors.

**FIGURE 4:**
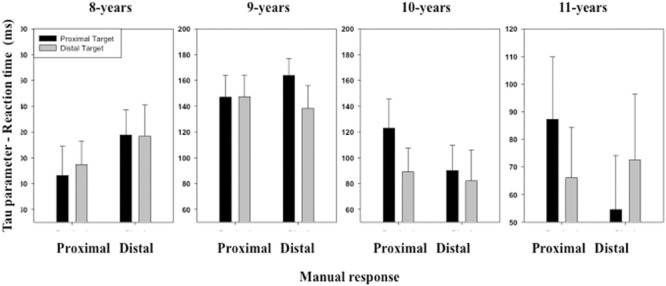
**Mean τ parameter of the ex-Gaussian distribution (RT) as a function of Age, Target location (proximal, distal), and Response (proximal, distal).** Error bars represent standard errors.

The ANOVA performed on the σ and τ parameters of the ex-Gaussian distribution of RT distribution revealed no significant effect of either the main factors or the interactions between the main factors (see **Figures 3** and **4**).

### Movement Time

**FIGURE 5:**
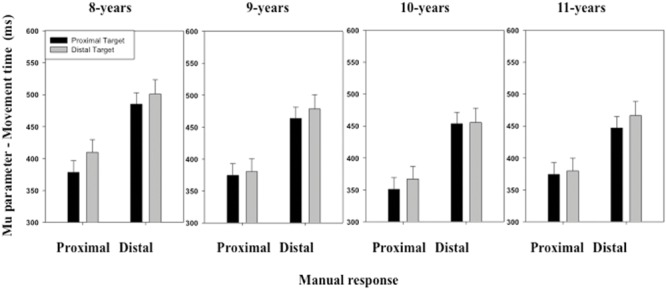
**Mean μ parameter of the ex-Gaussian distribution (movement time, MT) as a function of Age, Target location (proximal, distal), and Response (proximal, distal).** Error bars represent standard errors.

The ANOVA performed on the μ parameter of the ex-Gaussian distribution of MT distribution only revealed a main effect of *Stimulus location* [*F*(1,116) = 7.34, *p* < 0.01] and *Manual response* [*F*(1,116) = 246.43, *p* < 0.01] (see **Figure 5**). Overall, MTs were shorter when the stimulus was displayed at a proximal (*M* = 415 ms, *SD* = 108 ms) than a distal location (*M* = 429 ms, *SD* = 123 ms). MTs were also shorter when the children produced a proximal (*M* = 376 ms, *SD* = 103 ms) than a distal response (*M* = 468 ms, *SD* = 110 ms). The interactions between these two factors and between the last two factors and *Age group* were not significant.

**FIGURE 6:**
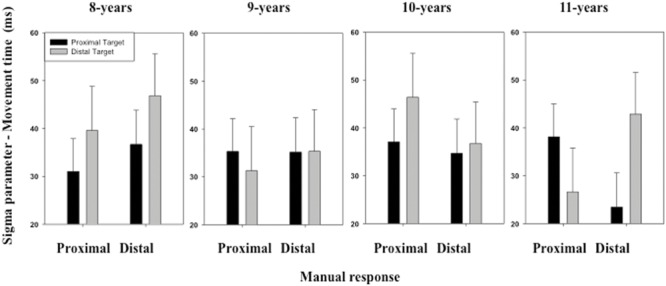
**Mean σ parameter of the ex-Gaussian distribution (MT) as a function of Age, Target location (proximal, distal), and Response (proximal, distal).** Error bars represent standard errors.

The ANOVA performed on the σ parameter of the ex-Gaussian distribution of MT distribution revealed no significant effect of either the main factors or the interactions between the main factors (see **Figure 6**).

**FIGURE 7:**
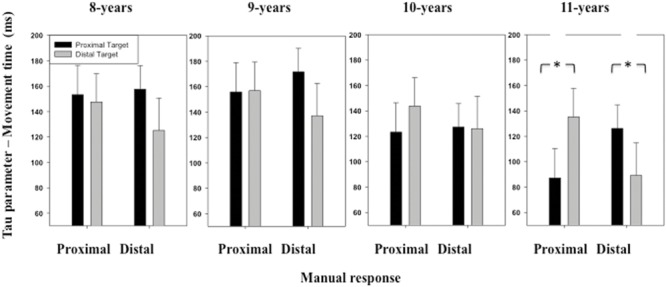
**Mean τ parameter of the ex-Gaussian distribution (MT) as a function of Age, Target location (proximal, distal), and Response (proximal, distal).** Error bars represent standard errors. The stars indicate significant differences.

The ANOVA performed on the τ parameter of the ex-Gaussian distribution of MT distribution revealed a significant interaction between *Stimulus location* and *Manual response* [*F*(1,116) = 7.59, *p* < 0.01] (see **Figure 7**). When the participants executed a proximal response, they produced a smaller amount of long responses when the stimulus was displayed at a proximal (*M* = 129 ms, *SD* = 129 ms) than a distal location (*M* = 145 ms, *SD* = 120 ms). When the participants executed a distal response, they produced a smaller amount of long responses when the stimulus was displayed at a distal (*M* = 119 ms, *SD* = 139 ms) than a proximal location (*M* = 145 ms, *SD* = 102 ms). Although the interaction between these last two factors and *Age group* was not significant, significant differences between congruent and incongruent conditions were observed for the 11 years children only, when responding to the proximal [*t*(29) = 1.87, *p* = 0.03] or distal [*t*(29) = 2.70, *p* < 0.01] location.

## Discussion

The main objective of the present study was to explore the effect of SRC over the period of age between 8 and 11-years. Overall, the data show that children had better performances for congruent than incongruent trials. In particular, despite the task required to process stimulus color only, RTs were shorter when producing a proximal response in the presence of a proximal stimulus and a distal response in the presence of a distal stimulus, than a proximal response in the presence of a distal stimulus or vice-versa. This effect corresponds to the classical SRC effect and was observed for the μ parameter (mean performance) but not for the σ (performance variability) and τ (asymmetry in the distribution) parameters of the ex-Gaussian distribution of RT distribution. However, the group analysis revealed that this pattern of results was affected by children age as only the 11-years children showed the typical pattern of SRC effect usually observed in adults (Tucker and Ellis, 2001; Olivier, 2006; Buetti and Kerzel, 2008; Coutte et al., 2015). SRC effect seems thus to appear at a specific period of children development, with a shift in the performance occurring between 8 and 11 years, as suggested by the data on RT (we indeed observed a trend toward significant effect for 10-years-old children). The absence of main effect of stimulus location or manual response on RTs suggested that the task difficulty and stimuli visibility were homogeneous across all the conditions and for all age groups. This is confirmed by the lack of effect of congruent-incongruent conditions on the σ and τ parameters of the ex-Gaussian fit procedure of the data relating to RT. Interestingly, 11 years old children produced fewer long-lasting movements in the congruent than the incongruent conditions (τ parameter of the ex-Gaussian function for MT). They indeed show a broader amount of long-lasting movements when performing a short movement in the presence of a distal target and when performing a long movement in the presence of a proximal target. This suggests that SRC effect evidenced from the age of 11-years in the RT data, also influences movement execution with a higher rate of long-lasting movement when there was no congruence between the location of a visual target and the extent of arm movement.

As a whole, the present findings are thus compatible with the idea of a critical period of qualitative change between the age of 8–10 years, in particular in the way visual information is processed in relation to other sensory systems (Gori et al., 2008) and the motor system (Adalbjornsson et al., 2008; Thibaut and Toussaint, 2010; Ego et al., 2016). The presence of the SRC effect may indeed be interpreted as the effect of predictive mechanisms associated with the perceptual-motor coupling linked to the processing of the visual stimulus (Hommel, 2011; Coutte et al., 2015). According to SRC effect, in the presence of a visual stimulus intrinsic and extrinsic characteristics of that stimulus are automatically processed, as well as the motor response aligned with the distance of the stimulus. Such an automatic motor coding of visual stimulus has been demonstrated in the past using either passive visual observation (Quinlan and Culham, 2007; Proverbio, 2012), visual discrimination task (Tucker and Ellis, 1998), visually triggered motor actions (Costantini et al., 2010) or reachability judgment tasks (Wamain et al., 2015). It has also been found for various spatial dimensions including right-left (Kornblum, 1994; Hommel, 2011) and up–down dimension (Cho and Proctor, 2002; Meiran, 2005). The pattern of results observed in the 11-years old children is thus compatible with the theory of event coding (Hommel et al., 2001), which argues that stimulus and response coding are not independent but share a common level of processing. The present findings are also compatible with Kornblum’s dual route model (Kornblum et al., 1990; Kornblum, 1994), which postulates independent responses selection depending on the stimulus attributes, which could nonetheless overlap in time depending on the one hand on the stimulus features automatically processed and, on the other hand, on the stimulus features intentionally linked to the task requirements. The effect found on RTs measurements may thus reflect the conflict between relevant and irrelevant features influencing response selection: a faster response initiation is indeed observed for the 11-years old in the congruent trials in comparison to incongruent ones.

Surprisingly, no SRC effect was found on MT (mu parameter of the ex-Gaussian function for MT) at all the ages tested in the present experiment. It was indeed reported in previous studies that SRC effect affects both RT and MT (Buetti and Kerzel, 2009; Coutte et al., 2015). According to Buetti and Kerzel (2009), SRC tasks affect the selection of manual responses during both motor preparation and execution stages, such that SRC effect is observed on RT (latency of response initiation), MT (duration of response execution) and movement kinematic parameters (initial direction of the response for instance). When stress is put on movement initiation (following instructions), SRC effect influences MT but not RT. By contrast, response precueing reduces SRC effect on movement parameters, while still preserving SRC effect on RT. As a whole, these results suggested that temporal characteristics of SRC effect reflect the dynamical properties of the planning and execution components of the manual motor responses (Buetti and Kerzel, 2009). The lack of effect of SRC in the present study on mean MT (mu parameter) could thus be attributed to the lack of strict constraint on the organization of the motor response. Because the instructions given to the children did not encourage short RTs, the conflict between target distance and movement amplitude could be resolved before movement initiation, affecting thus essentially RT. Indeed, in order to produce the correct response in the incongruent trials, the automatically activated motor response needed to be inhibited (Kornblum, 1994), at least in the 11 years-old children. According to Buetti and Kerzel (2008), this inhibition may be not dependent on an all-or-none process but may linger in the system depending on processing time and affect motor execution in the case of fast initiated responses, which was not the case in the present study (see also Coutte et al., 2015). However, MT distribution was asymmetric toward long-lasting movements in the incongruent compared to the congruent spatial conditions in the 11 years old children (τ parameter of the ex-Gaussian function), suggesting that SRC effect slightly modulated movement execution in the oldest children.

With respect to the developmental trajectory of the SRC effect, the data in the present study suggest then that the two processing routes are not available in younger children, or at least not fully functional. Indeed, the lack of SRC effect on RT before the age of 10 years suggests an absence of automatic motor activation associated with the perception of the irrelevant spatial properties of the stimulus. It seems then that younger children refer to the visual features of the stimulus but fail to integrate multi-modal and motor related information associated with that stimulus. This interpretation is in line with the demonstration by Gori et al. (2008) that it is not until the age of 8–10-years that multimodal information is integrated in an optimal fashion. Typically, younger children succeed in perceptual tasks by selecting the most appropriate sensory channel (e.g., visual or haptic information) instead of considering more cues and integrate them, as older children do, to comply with the constraints of the task. This interpretation is also supported by the finding that the period between 8–10-years is characterized by improvements in sensorimotor integration and the development of motor predictive models making possible the response to new or fickle environmental constraints (Jansen-Osman et al., 2002; Gori et al., 2008; Thibaut and Toussaint, 2010).

To summarize, the present study investigated the developmental aspect of the conflict inherent to the processing of relevant and irrelevant features of a visual stimulus during response selection and execution in a classical SRC task. We found that mainly RT was affected by the incongruence between instruction-dependent and motor related visual information. Furthermore, the effect of the conflict between the different features of the visual stimulus appears only in children older than 10 years. Accordingly, the present results provide additional evidence for a reorganization of the perceptual system integrating multimodal information in a particular period of development and preparing more efficiently the body to act in the environment. Further investigations would be important to evaluate, through different experimental constraints and finer measures of motor responses (e.g., movement kinematics), how RTs and MTs are affected in SRC task in childhood. This would provide valuable information on the improvement of the sensory, motor and cognitive mechanisms supporting response selection, planning and execution at major stages of the children development.

## Author Contributions

AR organized the experiment, tested the participants, analyzed the data, wrote the paper. GO organized the experiment, wrote the paper. YC organized the experiment, analyzed the data, wrote the paper.

## Conflict of Interest Statement

The authors declare that the research was conducted in the absence of any commercial or financial relationships that could be construed as a potential conflict of interest.
